# Hybrid PET/MRI of large vessel vasculitis: Radiation dose compared to PET/CT with view on cumulative effective dose

**DOI:** 10.1007/s00508-024-02336-2

**Published:** 2024-03-08

**Authors:** Johanna-Felicia Brauner, Sazan Rasul, Dominik Berzaczy, Daniela Beitzke, Tim Wollenweber, Dietrich Beitzke

**Affiliations:** 1 Department of Biomedical Imaging and Image-guided Therapy, Medical University of Vienna, Währinger Gürtel 18–20, 1090 Vienna, Austria; 2 University for Applied Science Wiener Neustadt, Johannes Gutenberg-Straße 3, 2700 Wiener Neustadt, Austria

**Keywords:** Aortitis, Vasculitis, Positron emission tomography computed tomography, Positron emission tomography magnetic resonance imaging, Inflammation

## Abstract

**Background:**

The diagnosis of large vessel vasculitis (LVV) is often challenging due to the various clinical appearances and the low prevalence. Hybrid imaging by positron emission tomography and computed tomography (PET/CT) is a highly relevant imaging modality for diagnostics and disease surveillance but may be associated with a significant amount of radiation dose especially in patients with complications.

**Objective:**

The aim of this retrospective analysis was to compare the image quality and impact of hybrid imaging methods PET/CT and PET/MRI on the potential for dose reduction.

**Methods:**

This retrospective single-center study included a cohort of 32 patients who were referred to PET/MRI for the evaluation of LVV, including graft infections and fever of unknown origin. This cohort was compared to a similar cohort of 37 patients who were examined with PET/CT in the same period. Mean radiation dose as well as image quality to establish a diagnosis were compared between the groups.

**Results:**

The mean radiation dose applied in PET/MRI was significantly lower when compared to PET/CT (mean 6.6 mSV vs. 31.7 mSV; *p* < 0.001). This effect was based on the partially multiphasic CT protocols. At the same time, diagnostic image quality using a 4-point scale showed similar results for both imaging modalities in the work-up of LVV.

**Conclusion:**

With PET/MRI, the radiation exposure can be significantly reduced with similar image quality and diagnostic impact. Patients with LVV have a higher risk of receiving a clinically relevant cumulative effective dose (CED) and PET/MRI should be made available to them.

## Introduction

In large vessel vasculitis (LVV) symptoms are diverse and sometimes nonspecific. Both clinical and laboratory parameters may be variable and nonspecific, often leading to a delay in an exact diagnosis [1]. Complications of LVV include rupture, thrombus formation and ischemic events [2]. Infectious aortitis may cause sepsis and death if not adequately therapeutically addressed [3].

Early diagnosis and treatment initiation are therefore of tremendous importance to avoid complications. In addition, LVV often requires follow-up under treatment to monitor treatment success [4]. Clinical work-up for the evaluation of suspected LVV includes the need for appropriate imaging techniques to locate and quantify the degree of vascular inflammation. Ideally, the chosen technique should be suitable for treatment monitoring too.

Currently, hybrid computed tomography and positron emission tomography (PET/CT) is the preferred modality due to its high sensitivity (79–93%) and specificity (64–91%) for the detection as well as the follow-up examinations for LVV [5]. With this modality, inflammatory activity (with PET) and screening for vascular complications (with CT or integrated CT angiography) can be diagnosed [6, 7].

As LVV occurs more frequently in the young female population, it is important to lower radiation dose with every single examination to keep the cumulative radiation dose as low as possible while maintaining diagnostic confidence [2, 8]. According to the current German guidelines on the management of LVV, [^18^F]FDG PET/CT represents the gold standard for diagnosing LVV [9].

The commonly used tracer for the PET component is fluorodeoxyglucose ^18^F ([^18^F]FDG), due to the affinity of glucose for participation in metabolically active processes, as seen in inflammatory tissues in LVV [2, 10] and [^18^F]FDG PET/CT has proved its usefulness and robustness; however, the main drawback of the method is the radiation exposure, with values of 21.64 ± 5.20 mSv reported in the literature [11].

Combined PET/MRI might become an attractive alternative to PET/CT with a great potential to reduce the radiation exposure associated with hybrid imaging At present [^18^F]FDG PET/MRI is mostly used for diagnostic imaging and follow-up of oncological diseases; however, the areas of application have already been extended to cardiovascular imaging [12]. The integrated MR component offers the possibility to provide detailed anatomical and morphological information about inflammatory diseases of the aorta, without the radiation dose concerns caused by the CT component. The advantages of MRI are the excellent soft tissue characterization of the vessel wall, without exposing the patient to radiation. The aim of this study was to compare the image quality and impact of hybrid PET-MRI and PET-CT with respect to the potential for dose reduction.

## Material and methods

### Patients

This retrospecitve single-center analysis included 68 patients who underwent clinically indicated [^18^F]FDG PET/MRI or [^18^F]FDG PET/CT for the evaluation of suspected or already known LVV between April 2014 and June 2018. For data collection, referrals to total 36 [^18^F]FDG PET/MR and total 38 [^18^F]FDG PET/CT examinations were screened for inflammation of the large vessels, including LVV (*n* = 61), fever of unknown origin (FUO) (*n* = 4) and suspected graft infections (*n* = 9).

**Fig. 1:**
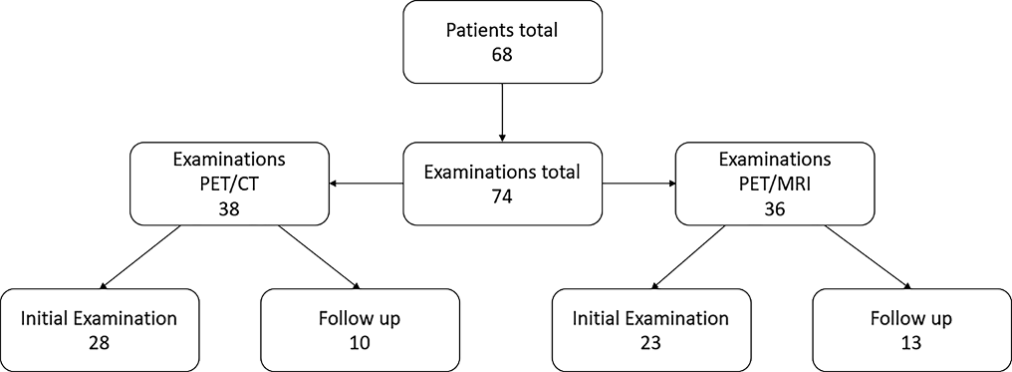
Flowchart of retrospective data analysis

Overall 74 examinations were analyzed in the study with 51 baseline examinations (23 [^18^F]FDG PET/MRI and 28 [^18^F]FDG PET/CT) and 23 follow-up examinations under treatment or for disease control (13 [^18^F]FDG PET/MRI and 10 [^18^F]FDGPET/CT). Details are shown in Fig. 1.

Repetitive studies were observed in this analysis. In this study two patients underwent two [^18^F]FDG PET/MRI, one patient three [^18^F]FDG PET/MRI and one patient one [^18^F]FDG PET/MRI as well as two [^18^F]FDG PET/CT examinations. Follow-up examinations included stent graft infections (*n* = 2), inflammatory aortic aneurysms (*n* = 3), Takayasu arteritis (*n* = 7), Ormond’s disease (*n* = 5), giant cell arteritis (*n* = 3) and nonspecified vasculitis (*n* = 3).

If the application of contrast medium in CT or MR was contraindicated or there was reduced renal function (eGFR ≤ 30 ml/min), images without contrast were acquired in both methods. The analysis was approved by the local ethics committee (EK 1795/2018).

### Patient preparation

All patients had to fast at least 5h, while drinking still water. Blood sugar levels were checked to rule out hyperglycemia prior to imaging. An intravenous administration of 245 ± 25.6 MBq [18F]FDG for PET/MRI and 280 ± 30.3 MBq [18F]FDG for PET/CT followed.

### PET/MRI protocol

The [^18^F]FDG PET/MRI was performed using a hybrid PET/MR system (Biograph mMR, Siemens-Healthineers, Erlangen, Germany) with a PET integrated into a 3T MR system. Of the participants of the PET/MRI cohort 30 (93.75%) received 0.15 ml/kg body weight gadobutrol (Gadovist®, Bayer Schering, Austria) injected as a contrast medium. The images were acquired in 4–5 bed positions with a 5-min acquisition time per bed position. The imaging protocol consisted of a contrast-enhanced magnetic resonance angiography of the thoracoabdominal aorta with a bolus tracking technique during the administration of gadobutrol, with subtraction technology and T2 whole-body STIR and T1 for attenuation correction sequences before and after the administration of contrast medium. Of the patients two underwent PET/MRI without contrast due to renal failure.

### PET/CT protocol

The [^18^F]FDG PET/CT was performed on a 64-row hybrid PET/CT (Biograph TruePoint 64; Siemens Healthineers) and 32 participants (88.89%) of the PET/CT cohort received a contrast agent injection (1.5 ml/kg body weight iomepro; Iomeron® 300, Bracco Austria). Due to a known allergy to the contrast media or poor renal function, four persons of the PET/CT cohort underwent a PET/CT without contrast. There were 39.47% of CT scans performed in the arterial and venous phases (either CT angiography of the thoracic aorta or the abdominal aorta). Only a venous phase was acquired in 50%, and 10.53% of the examination were performed as a low-dose scan without contrast.

### Estimation of radiation dose

To compare the radiation dose of PET/CT and [^18^F]FDG PET/MRI, the applied values of the activity of [^18^F]FDG PET in MBq and the radiation dose of CT in DLP were converted into effected dose and reported in mSv. In the PET component, the weighting factor 0.027 mSv/MBq, which corresponded to the conversion value IRCP publication, was used to convert the tracer activity from MBq to mSV [13]. The weighting factor 0.015 mSv/(mGy*cm) was used according to the American Association of Physisits in Medicine Report°N096 [14] to convert the dose-length product in mGy*cm to the effective radiation dose in mSv.

### Diagnostic image quality

The classification of diagnostic image quality was graded in categories from 0 to 3. For both modalities, particular emphasis was placed in the evaluation on the assessability of the soft tissue and an artifact-free and accurate superimposition of the hybrid images. The evaluation was independently performed by 2 radiologists with more than 10 years of experience in vascular imaging.

Images in category 0 were rated as nondiagnostic, because the insufficient image quality could not be used for a reliable diagnosis of LVV. Category 1 images were rated as diagnostic, although the image quality was still reduced. Images in category 2, however, were of good quality and category 3 images were of excellent quality.

### Statistical analysis

Statistical analyses were performed using Microsoft Excel. Continuous results are given as mean ± standard deviation and range.

To compare effective radiation dose from [^18^F]FDG PET/MRI and [^18^F]FDG PET/CT, a t-test for unpaired samples with unequal variances was performed with a significance level at *p* < 0.001. The null hypothesis of a uniform variance of the parameter effective radiation dose was previously rejected by performing Levene’s test.

For the evaluation of diagnostic image quality, the interrater agreement was checked using Cohen’s kappa. For the ordinal variable image quality/diagnostic confidence a Mann-Whitney U‑test was performed for which a significance level of α ≤ 0.01 was determined.

## Results

### Patients

The parameters observed among the patients were age, body mass index (BMI), and blood glucose level before injection of the tracer [18F]FDG (Table 1). There was no significant difference between the patient groups regarding these parameters.

**Table 1:** Study population and imaging diagnoses

Characteristics of the study population		
*Variables*	*PET/MRI*	*PET/CT*
Gender (number)	Female (11)	Female (20)
	Male (21)	Male (17)
Age (years)	55 ± 17 [25–87]	58 ± 14 [24–78]
BMI (kg/m^2^)	26 ± 4.2 [19–36]	26 ± 5.6 [17–46]
Blood glucose level before injection of tracer (mg/dl)	107 ± 17 [65–145]	114 ± 18 [76–163]
*Diagnoses*	*PET/MRI (N)*	*PET/CT (N)*
No active aortitis total	21	29
Active aortitis total	15	9
Remission after active aortitis	6	5
Active aortitis, not classified	5	6
Inflammatory aortic aneurysm	5	0
Takayasu’s arteritis	4	3
Graft infection	4	0
Retroperitoneal fibrosis	3	2

Of the 74 total examinations, 24 revealed active aortitis, (15 on [^18^F]FDG PET/MRI and 9 on [^18^F]FDG PET/CT). In the remaining 50 examinations, active vascular inflammation could be ruled out (21 using [^18^F]FDG PET/MRI and 29 using [^18^F]FDG PET/CT). It should be noted that in the follow-up examinations a total of 11 remissions of previously diagnosed LVV under treatment was recorded (6 on [^18^F]FDG PET/MRI and 5 on [^18^F]FDG PET/CT). Details are shown in Table 1.

### Effective dose

The effective radiation dose of the PET/MRI cohort per examination was significantly lower (*p* ≤ 0.05) than the PET/CT cohort per examination as shown in Table 2.

**Table 2:** Effective radiation dose, with results listed by imaging components

Effective radiation dose			
	mSv	MBq	mGy*cm
*PET/MR*	6.56 ± 0.68 [5.31–7.88]	247.06 ± 25.09 [196.5–292]	–
*PET/CT*	31.69 ± 9.12 [12.89–54.33]	–	–
PET component	7.57 ± 0.83 [6.37–9.72]	280.37 ± 30.74 [235.93–360]	–
CT component	25.26 ± 8.87 [14.46–50.42]	–	1684 ± 591.33 [964–3361]

A t-test for unpaired samples with unequal variances with a significance level at *p* < 0.001 showed that there was no significant difference between the radiation dose of PET (MBq) components viewed alone.

**Fig. 2:**
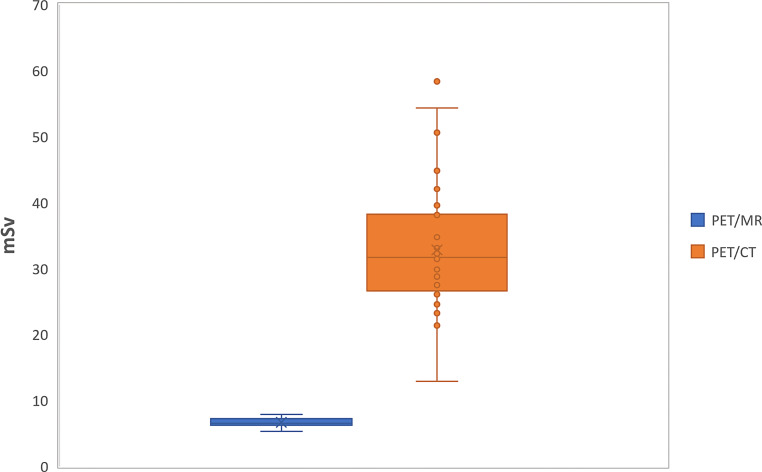
Boxplot of effective radiation dose Illustration of median, mean, upper and lower quartile, upper and lower whisker in both modalities

Of the patients of the PET/CT cohort three received a radiation dose higher than 50 mSv for one examination. Regarding the cumulative dose for each patient there was no patient of the PET/MRI cohort who received repetitive [^18^F]FDG PET/MRI who was exposed to 50 mSv or more. One patient who underwent two [^18^F]FDG PET/CT and one [^18^F]FDG PET/MRI examinations during the period of this analysis was exposed to a cumulative radiation dose of 92.23 mSv. None of the patients had a cumulative dose of 100 mSv or above. The results of the main parameter effective dose are shown in Table 2 and Fig. 2.

### Image quality

**Fig. 3:**
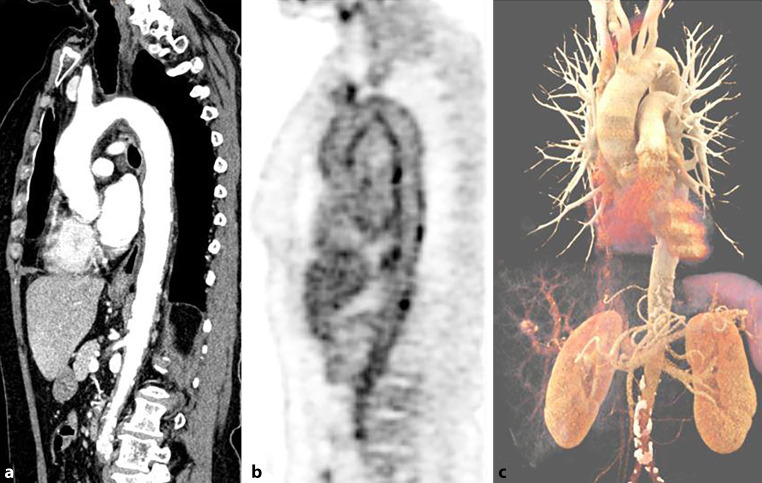
[^18^F]FDG PET/CT image showing an active aortitis with vessel wall thickening in sagittal reformatted CT angiography images (**a**) and [^18^F]FDG uptake at the aortic arch and the descending aorta (**b**). 3D volume rendering of the aorta shows no pathologic dilatation of the vessel (**c**). The tracer activity of 263MBq corresponding to 7.1 mSv and a DLP of 1625 mGy*cm corresponded to 24.375 mSv, and made up a total effective dose of 31.5 mSv

**Fig. 4:**
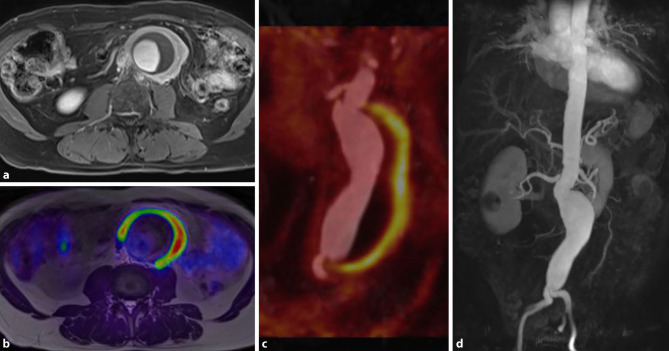
[^18^F]FDG PET/MR image of an inflammatory aortic aneurysm. Axial T1 vibe image after contrast shows the abdominal aortic aneurysm with vessel wall thickening and intraluminal thrombus formation (**a**). Fused images show pronounced [^18^F]FDG uptake within the thickened vessel wall as well (**b**, **c**). 3D rendering of the MR angiography shows the localized widening of the infrarenal aorta (**d**). The tracer activity of 289MBq corresponded to an effective dose of 7.8 mSv

The image quality of [^18^F]FDG PET/MRI was rated in all cases as good (category 2) or excellent image quality (category 3). In [^18^F]FDG PET/CT, only a single scan was rated as diagnostic but image quality reduced (category 1) (see Table 3). This rating was based on multiple motion and breathing artefacts during CT acquisition. The analysis of diagnostic confidence in image quality showed no significant difference between the two groups. There were moderate interobserver agreements for [^18^F]FDG PET/MRI (κ = 0.366) and [^18^F]FDG PET/CT (κ = 0.578) for image quality. Figures 3 and 4 demonstrate representative [^18^F]FDG PET/CT and [^18^F]FDG PET/MR images from this study.

**Table 3:** Results of image quality

Image quality								
Category	PET/MRI				PET/CT			
	Reader 1		Reader 2		Reader 1		Reader 2	
0–3	Number	Percent	Number	Percent	Number	Percent	Number	Percent
0	0	0	0	0	0	0	0	0
1	0	0	0	0	0	0	1	2.63
2	5	13.89	4	11.11	2	5.26	2	5.26
3	31	86.11	32	88.89	36	94.74	35	92.11

## Discussion

The main finding in this study was that [^18^F]FDG PET/MRI and [^18^F]FDG PET/CT are equally well suited as a diagnostic tool in the imaging work-up of LVV while [^18^F]FDG PET/MRI achieved significantly lower radiation doses.

Especially in diseases that require repetitive imaging, avoiding unnecessary radiation doses has become more important in medical imaging with the aim to reduce the cumulative effective dose. [15, 16].

For the work-up of LVV and FUO in our institution, the effective radiation dose per examination was 6.56 mSv, on average in the PET/MRI group, while it was 31.69 mSv, on average in the PET/CT group. This difference is mainly based on the lack of radiation exposure of the often multiphasic CT component; however, it should be noted that there are differences between the two modalities regarding the quantitative PET component. The MR images are not correlated with tissue attenuation coefficients as CT images are. The reliable determination of the tracer uptake in a PET/MRI requires an attenuation correction of the PET data, which calculates an attenuation based on soft tissue, bone, and hardware components.

Our analysis showed that about 78% of the radiation dose could be reduced while providing the same diagnostic quality in the evaluation of LVV. This is especially of importance when multiple examinations might become necessary in the follow-up of inflammatory LVV [17, 18]. A lower applied radiation dose in every single examination to minimize stochastic radiation effects from cumulative radiation dose might reduce the carcinogenic risk of imaging in this cohort of patients. In this retrospective study, 14 [^18^F]FDG PET/MRI and 8 [^18^F]FDG PET/CT examinations were performed on patients younger than 51 years of age. The increase in the risk of developing fatal cancer in this cohort is estimated at 5% per administered Sv for the general population. While this risk increases to 15% per Sv in children, it decreases to 1% per Sv in people in their seventh decade of life [19]. Especially for young patients, PET/MRI might be preferable because it is a lower-dose variant with at least the same diagnostic confidence as PET/CT.

Due to the often unclear clinical presentation, it is assumed that other radiological diagnostics have already taken place before the specific examination using PET/MRI or PET/CT. Because the CT is a readily available imaging technique that is firmly established in the diagnosis of a variety of diseases, there has been a dramatic increase in average patient radiation exposure since its invention. Therefore, it is even more important to be able to save radiation doses in the future [15, 20, 21].

The PET/MRI combines the strength of these two single modalities. The hybrid imaging method enables the acquisition of information about the severity of the inflammation (with PET) and about the anatomical changes via MRI [22]. It is a relatively novel imaging technique whose areas of application are currently expanding [23]. In imaging of LVV, not only is the extent of the inflammation of importance but also anatomical changes are decisive for diagnosis and treatment surveillance. Thus, not every imaging method is suitable to reflect both aspects of this disease. Previous studies have already suggested that hybrid imaging methods [^18^F]FDG PET/CT and [^18^F]FDG PET/MRI provide the most reliable information on both aspects. The [^18^F]FDG PET/CT has already been well studied in this application [24, 25]. A study by Clemente et al. examined childhood-onset Takayasu’s arteritis patients using [^18^F]FDG PET/MRI and compared cohorts with positive PET and positive MRA with positive PET but negative MRA. It was shown that the hybrid method added information about the extent of the inflammation with the PET component, which is not presented in every patient in an MRA examination alone [26].

Cerne et al. showed that PET/MRI delivers more information about inflammatory markers in the diagnosis and follow-up of LVV compared to laboratory findings because this imaging method characterizes the severity and the extent of the inflammation. Inflammatory markers proved to be too unspecific and, compared to PET/MRI do not allow a statement about anatomical changes and the extent of the inflammation [27].

Laurent et al. evaluated a correlation between the clinical and laboratory presentation of patients with Takayasu arteritis and giant cell arteritis, and the results of imaging using [^18^F]FDG PET/MRI. The image analysis was graded based on the tracer uptake in the vessels, which was compared to the tracer uptake in the liver, using grading from 0 to 3, and by measuring the thickening and enhancement of the aortic wall on delayed enhancement MR images. This study showed that the tracer uptake in the vessels correlated with the quantity of the inflammation, proven by the clinical and laboratory results. In conclusion, [^18^F]FDG PET/MRI might even be suitable to replace invasive methods such as biopsies of the temporal artery, or as a diagnostic tool for giant cell arteritis [28], and appears to be a suitable method for detection and follow-up examinations in LVV; however, that study did not contain any comparison to other imaging methods.

In another study, Einspieler et al. drew comparisons regarding visual and quantitative parameters between both hybrid-imaging methods, [^18^F]FDG PET/MRI and [^18^F]FDG PET/CT, in patients with known LVV. The image scoring revealed the intensity of the arterial uptake of the tracer in contrast to the uptake into the liver. The statistical analysis was made with the measurements SUV_max_ and the target to background ratio for each measured vascular segment. The calculation of the target background ratio SUV_max_ was divided by SUV_min_ using a region of interest in the blood pool. This study proved that both modalities are equivalent with respect to the visual scores compared to the measured inflammatory parameters [29].

In contrast to the studies listed above, initial diagnoses of LVV were included in this study. The data presented in our paper is in line with these publications showing that imaging-based diagnosis using [^18^F]FDG PET/MR appears to be possible at a low radiation dose in patients suspected of having LVV; however, previous studies did not compare [^18^F]FDG PET/CT vs. [^18^F]FDG PET/MR in terms of effective radiation dose of these two modalities. The present study was the first to show that [^18^F]FDG PET/MRI offers equivalent diagnostic image quality in the work-up of suspected inflammatory vascular disease when compared to [^18^F]FDG PET/CT with a relatively high sample size.

Current studies are researching new tracers that are intended to further improve the diagnosis and follow-up of LVV. As glucose acts in metabolically active processes, which occur physiologically to an increased extent in the brain and myocardium, the diagnosis of, for example, temporal arteritis or the assessment of the coronary arteries in LVV can be misleading. In addition, with the use of [^18^F]FDG it is not possible to distinguish between processes of active inflammation, chronic vascular remodeling, and residual disease activity after treatment. According to a study by Ćorović et al., the tracer [68Ga]-dotatate, which binds somatostatin receptor 2 (SSTR2), already used in neuroendocrine tumor diagnostics, seems promising. The SSTR2 is expressed by macrophages during inflammation and localizes around CD68+ macrophages, such as those found in inflamed carotid atherosclerotic plaques [30]. In addition, other promising tracers are currently being investigated with a view to expanding their areas of application. Some examples include [68Ga]-pentixafor, a PET tracer that was originally developed for cancer imaging by binding to CXCR4, which is expressed on several proinflammatory immune cell types, or 68Ga-FAPI, which binds to the transmembrane serine protease fibroblast activation protein‑α (FAP), whose expression is high in activated stromal fibroblasts at sites of tissue remodeling and is found primarily in pathological conditions such as fibrosis, scar/granulation tissue, cancer, and arthritis [31].

However, current limitations of PET/MRI include its limited availability worldwide and its often predominant use in the work-up and staging of oncologic disease. Furthermore, access to this modality is sometimes hindered by contraindications for MRI such as claustrophobia and non-MR-compatible implants or non-MR pacemaker systems; however, currently, even pacemakers are often deemed MR-compatible, which might improve access to PET/MRI in the future [32]. There are relative contraindications for contrast medium such as terminal kidney failure.

With respect to the investment costs and the ongoing operational expenses, PET/MRI is far more expensive than PET/CT. The costs are related not only to the price of a PET/MR hybrid device itself, but to the availability of the radiological staff that can operate this new device. There is the longer duration required for an MRI compared to a CT scan. For cancer patients, the costs of the routine staging or follow-up per examination is about 50% higher for PET/MRI than for PET/CT [33].

Although the cumulative radiation dose is a strong argument for establishing PET/MRI in the diagnosis of LVV, there are applications where PET/CT is more effective. PET/CT with CT angiography is advantageous for planning interventions in cases of complications like stenosis or aneurysm formation as a result of inflammation.

In terms of the radiation exposure derived from PET/CT, it should be noted that currently, a new generation of modern PET/CT scanners are entering the market. These scanners can provide whole-body scans with ultra-low dose (e.g., 0.37MBq/kg) protocols that show no significant difference in quantitative and qualitative image analysis [34]. These long axial field of view (LAFOV) PET/CT scanners show an increased signal-to-noise ratio and can achieve higher sensitivity based on a higher count density than the scanner used in our analysis. In addition, modern postprocessing methods, such as ultrahigh sensitivity (UHS) reconstruction modes on these new scanners, may cut the PET acquisition time in half, which would reduce the examination time [35]; however, to generate whole-body parametric PET images, a minimum of 45–60 min of scanning is required due to the slow kinetics of 18F-FDG.

### Limitations

The main limitation of this study is the small patient population and its retrospective nature in a single center. Different CT protocols were included in the PET/CT cohort, resulting in an elevated CT radiation dose when compared to CT scans without contrast alone. It should be noted that the PET/CT scanner used in this study is already outdated when compared with recent scanner technology. Additionally, a non-significantly higher 18F-FDG dose had to be applied in PET/CT when compared to PET/MRI as the PET component of the currently available PET/MRI system has a higher sensitivity.

In the risk-benefit balance, the radiation dose savings outweigh the limitations of PET/MRI. The disadvantages of PET/MRI, as with MRI, are its contraindications for use in individuals with claustrophobia, metallic implants, and non-MR-compatible pacemakers, Furthermore, the low availability due to the high costs currently limits the further spread of PET/MR [33].

Although comparable cohorts were considered in this study, both examinations were not performed on a single patient, which precludes exact comparability.

## Conclusion

In conclusion, [^18^F]FDG PET MRI might be an attractive alternative for the diagnostic work-up of LVV, with the advantage of a significantly lower radiation dose when compared to [^18^F]FDG PET/CT. The advantages of PET/MRI over PET/CT are based on significantly lower radiation exposure due to the lack of the radiation burden of the CT component. As diagnostic image quality is maintained, PET/MRI appears to be especially attractive for patients who require imaging follow-up.
